# Supplementary material for: Translational adaptation to heat stress is mediated by RNA 5‐methylcytosine in Caenorhabditis elegans
Source: EMBO J. 2020 Dec 7;40(6):e105496. doi: 10.15252/embj.2020105496 (PMC7957426; doi:10.15252/embj.2020105496)
Supplement: Supplementary file 1 — Appendix [file EMBJ-40-e105496-s001.pdf]

Appendix

Navarro et al., Translational adaption to heat stress is mediated by RNA 5-methylcytosine in *Caenorhabditis elegans*

Contents:

Appendix Figure S1
Appendix Figure S2
Appendix Figure S3
Appendix Figure S4

FIGURE S1

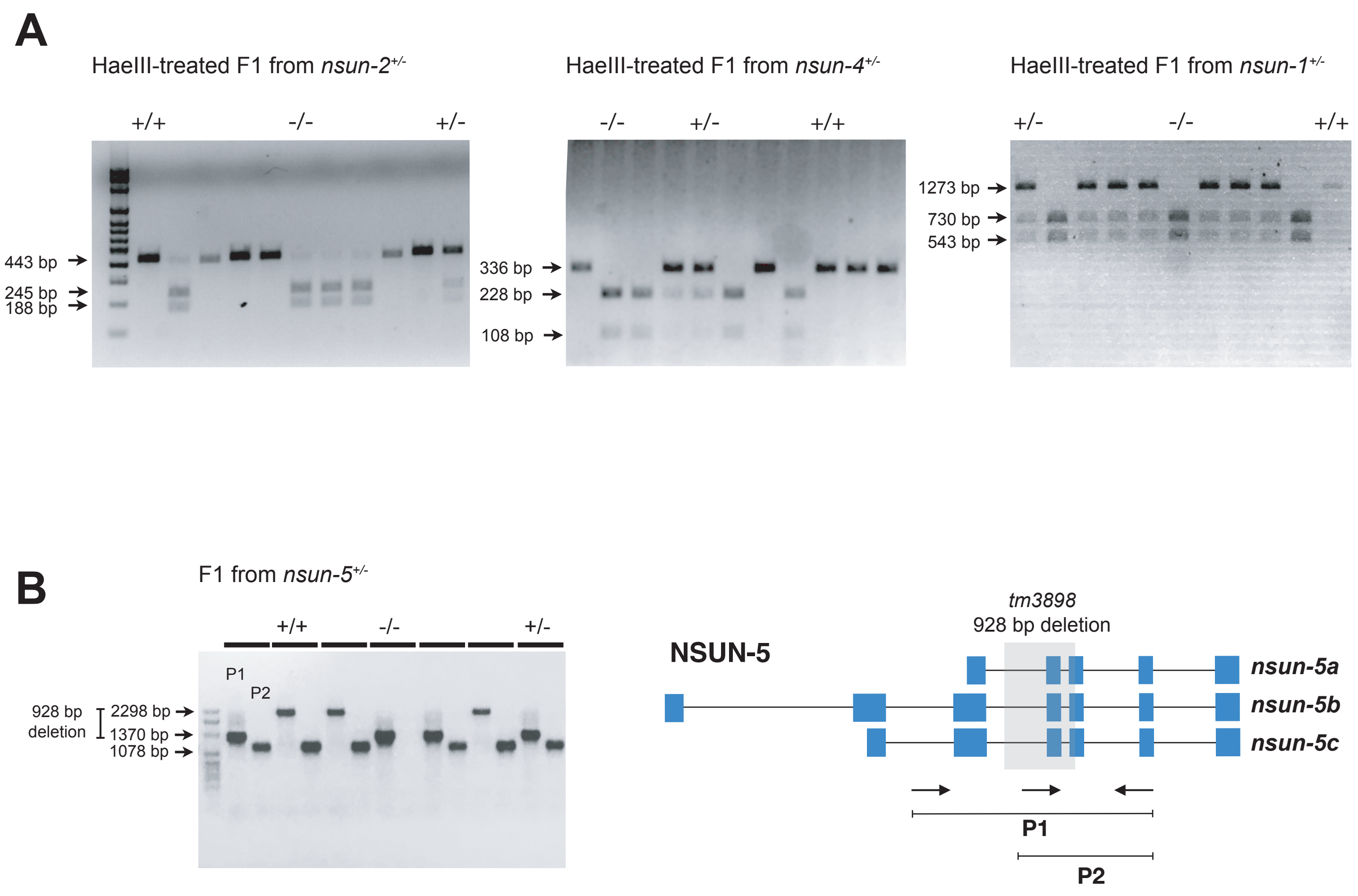

Figure S1 | Related to Figure 1. Genotyping of *nsun* mutants.
(A) HaeIII-treated DNA agarose gel showing genotypes of F1 individuals from heterozygous *nsun-1, nsun-2* and *nsun-4* mutants.
(B) DNA agarose gel showing genotypes of F1 individuals from a heterozygous *nsun-5* mutant; diagram showing primers used for genotyping of *nsun-5* mutation.

FIGURE S2

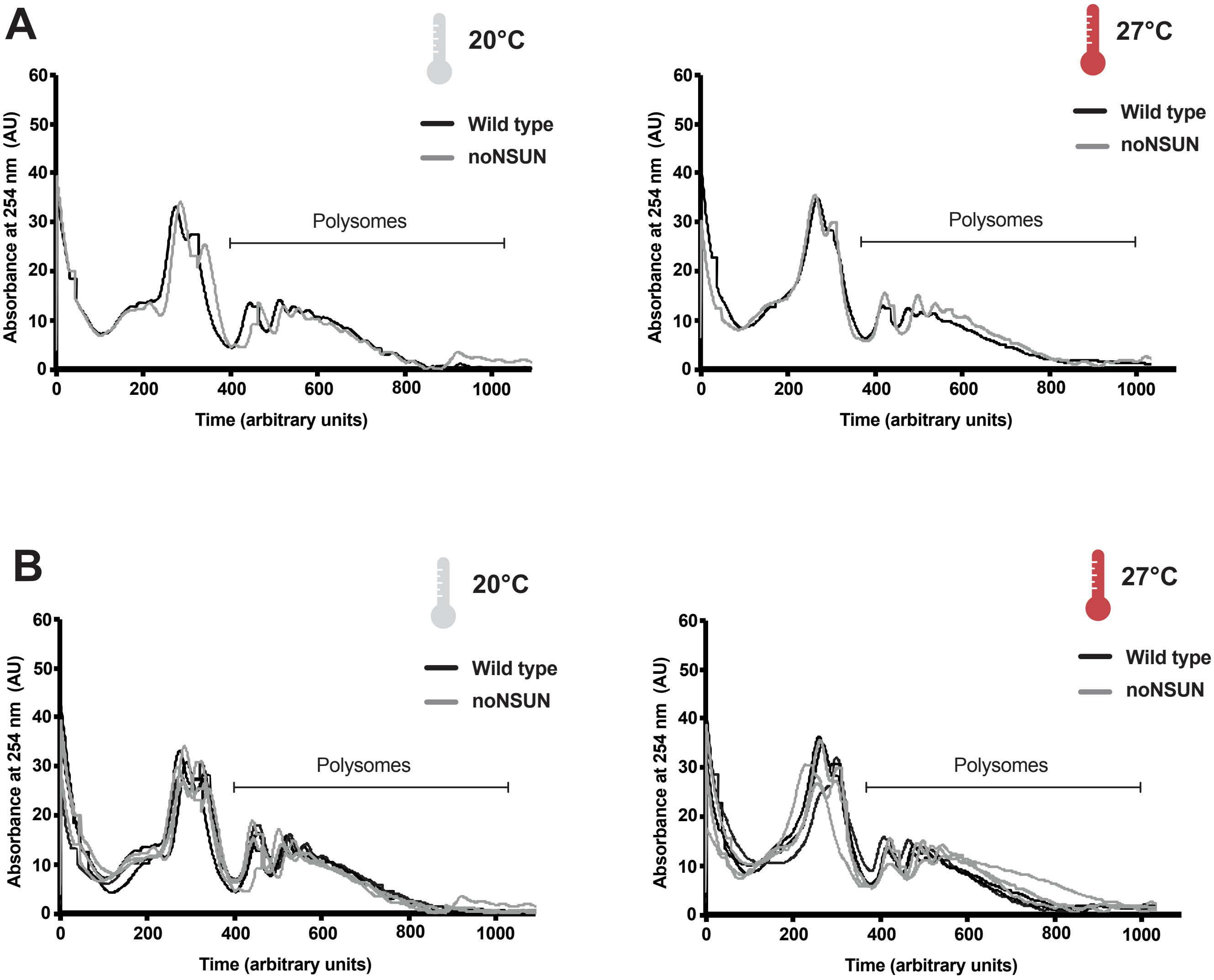

Figure S2 | Related to Figure 5A. Polysome profiles of wild type and noNSUN animals subjected or not to a heat shock at 27°C
(A) Representative polysome profiles of wild type and noNSUN strains at 20°C (left) and 27°C (right). Graphs normalised by the total area under the curve.
(B) Polysome profiles of wild type and noNSUN strains at 20°C (left) and 27°C (right) in triplicates. Graphs normalised by the total area under the curve.
Data information: n = 3 biological replicates.

FIGURE S3

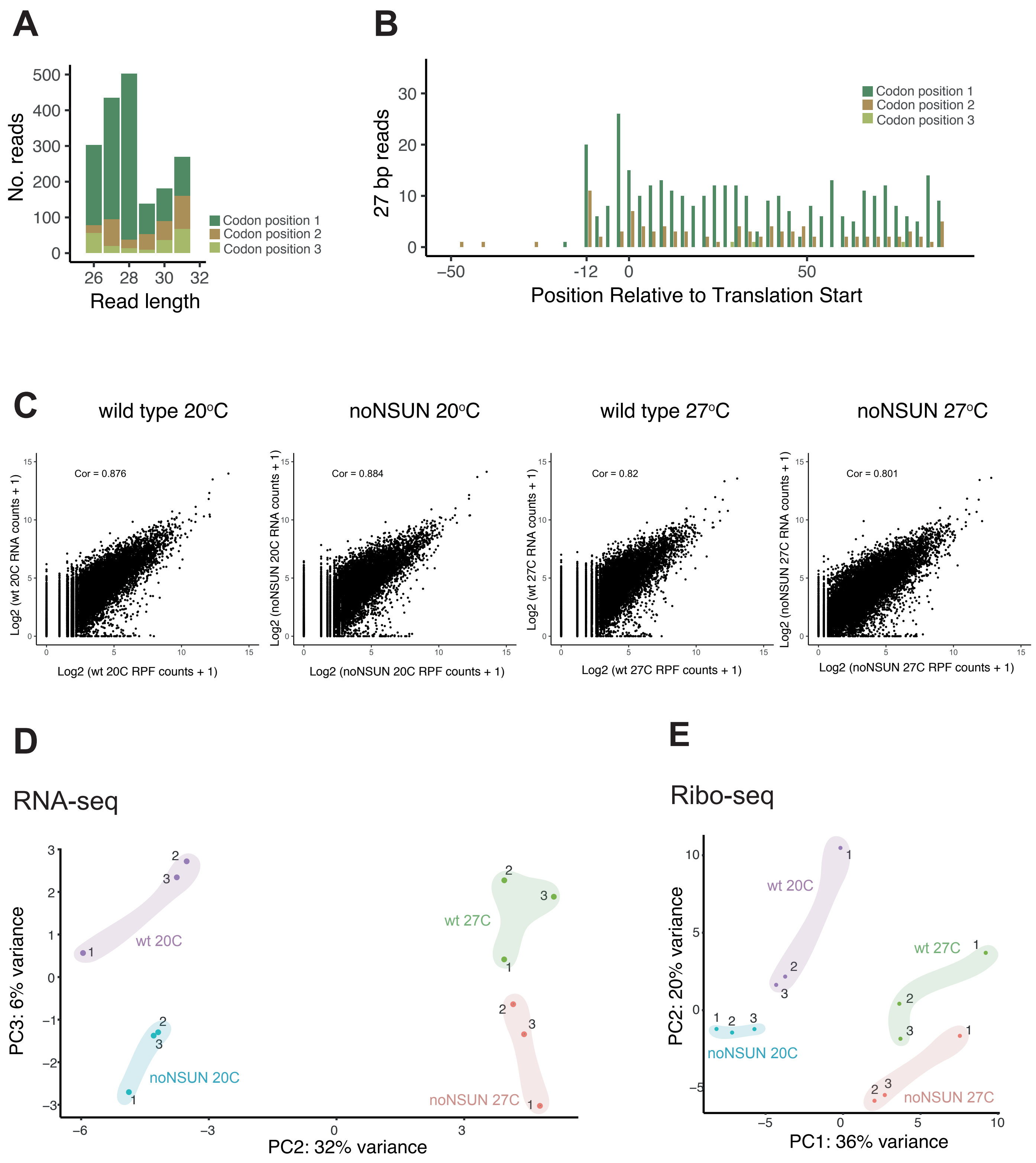

Figure S3 | Related to Figure 5. Quality assessment of ribosome profiling data.
(A) Number of reads aligning the to the CDS in each frame after length stratification. The frame of the 5′ nucleotide is shown.
(B) Representative meta-gene plot for 27 bp reads showing 3-nt periodicity. Number of reads for each codon position coloured according to the frame of their 5′ base relative to the CDS.
(C) Scatter plots showing the correlation between transcripts and footprints abundance for each gene at the indicated samples. Pearson correlation coefficient (r) is shown.
(D) PCA plot of RNA-seq counts for the 2000 genes with the highest variance.
(E) PCA plot of Ribo-seq counts for the 2000 genes with the highest variance.
Data information: n = 3 biological replicates.

FIGURE S4

A RNA-Seq

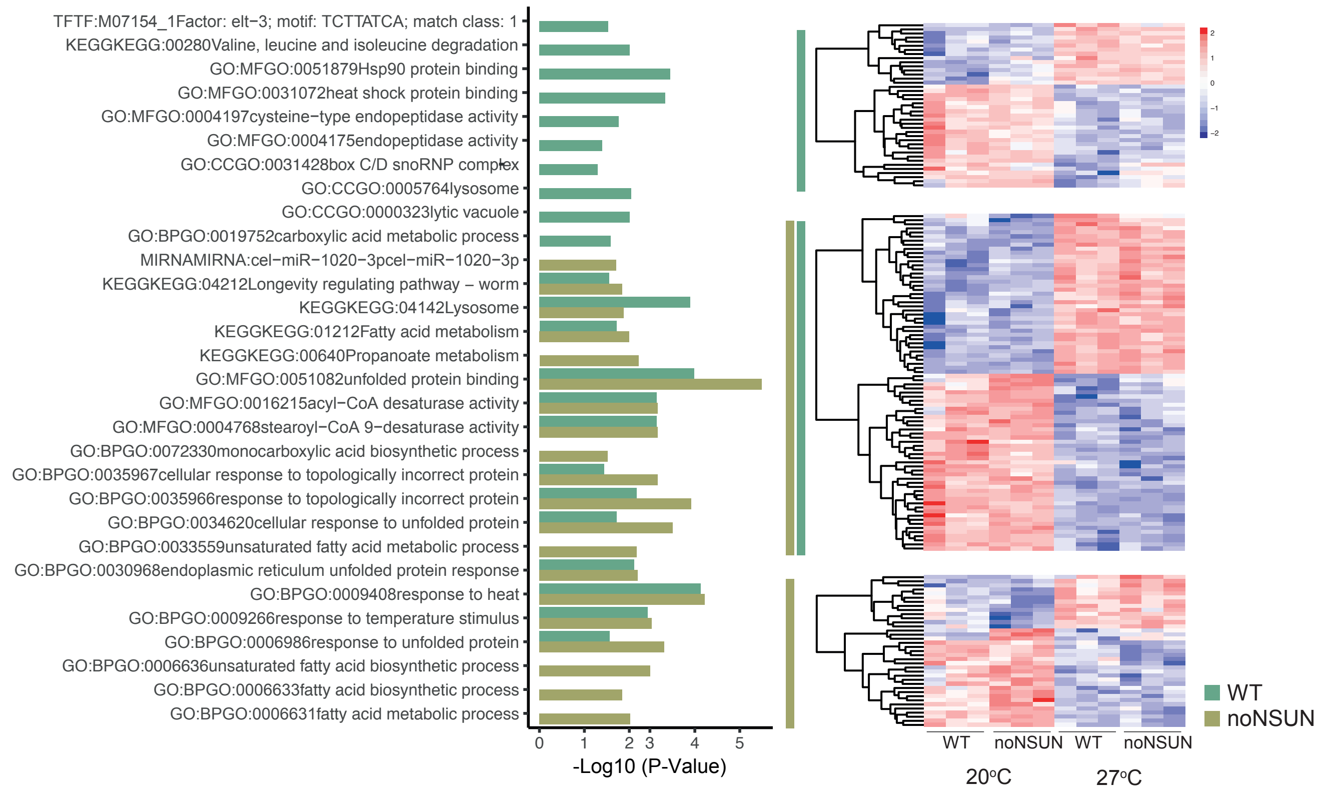

B Ribo-Seq

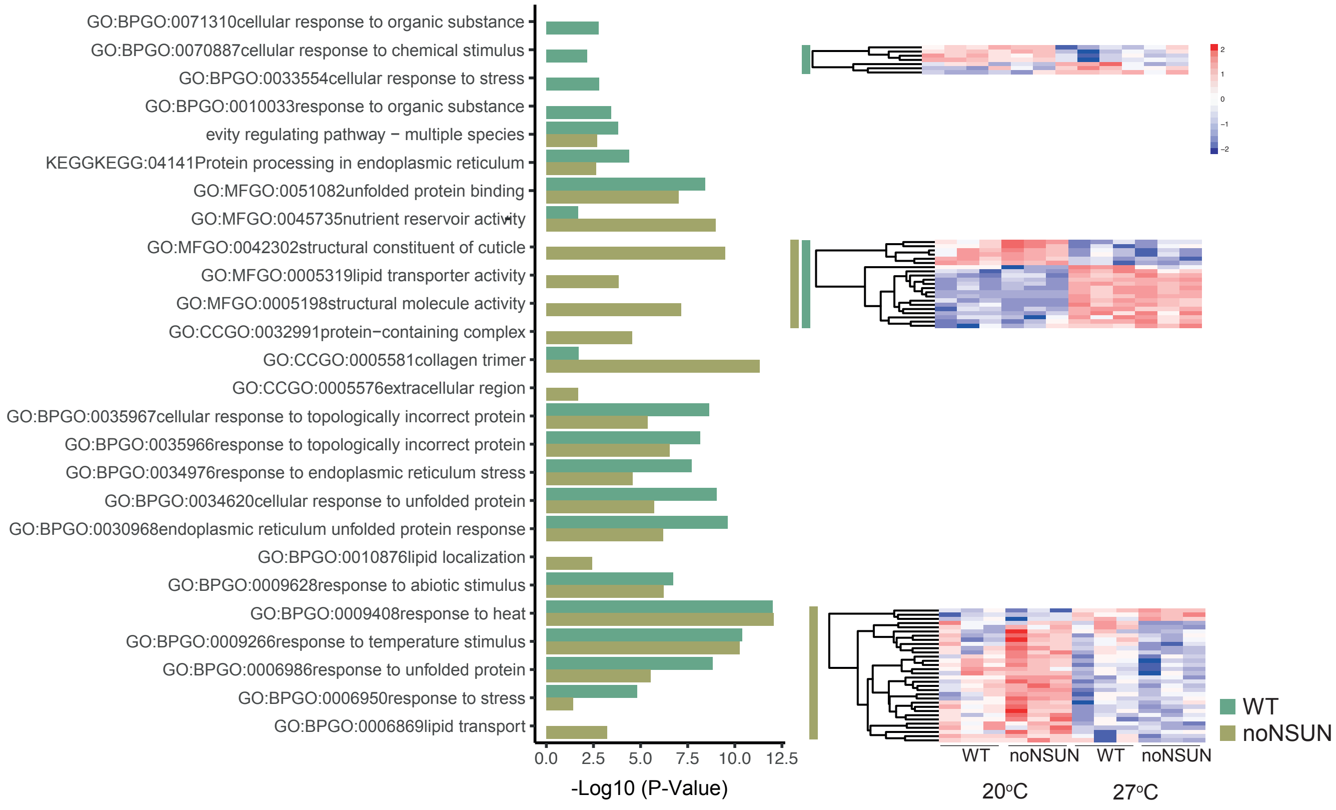

Figure S4 | Related to Figure 5. Differentially transcribed and translated genes in wild type and noNSUN strains upon heat shock. (A, B) Heatmaps and gene ontology enrichment (biological process) analysis for the comparison between 20°C and 27°C in the wild type and noNSUN samples. (A) shows RNA- seq (scaled normalised expression) and (B) shows Ribo-seq (scaled normalised RPFs). Data information: n = 3 biological replicates. WT = wild type.
