# Supplementary material for: Translational adaptation to heat stress is mediated by RNA 5‐methylcytosine in Caenorhabditis elegans
Source: EMBO J. 2020 Dec 7;40(6):e105496. doi: 10.15252/embj.2020105496 (PMC7957426; doi:10.15252/embj.2020105496)
Supplement: Supplementary file 2 — Expanded View Figures PDF [file EMBJ-40-e105496-s003.pdf]

Expanded View Figures

Figure EV1. Related to Fig 2. Enzymatic specificity of NSUN proteins in *C. elegans*.

A–C Determination of enzymatic specificity of 26S rRNA C2381 (A), 26S rRNA C2982 (B) and tRNA Leu-CAA C34 and C48 (C) methylation by targeted bisulphite-sequencing. Each column represents one cytosine in the sequence of interest; each line represents one clone sequenced. Enzymatic specificity of C2381 and C2982 in *C. elegans* has been independently demonstrated in other publications (Schosserer *et al*, 2015; Heissenberger *et al*, 2020).

© 2021 The Authors

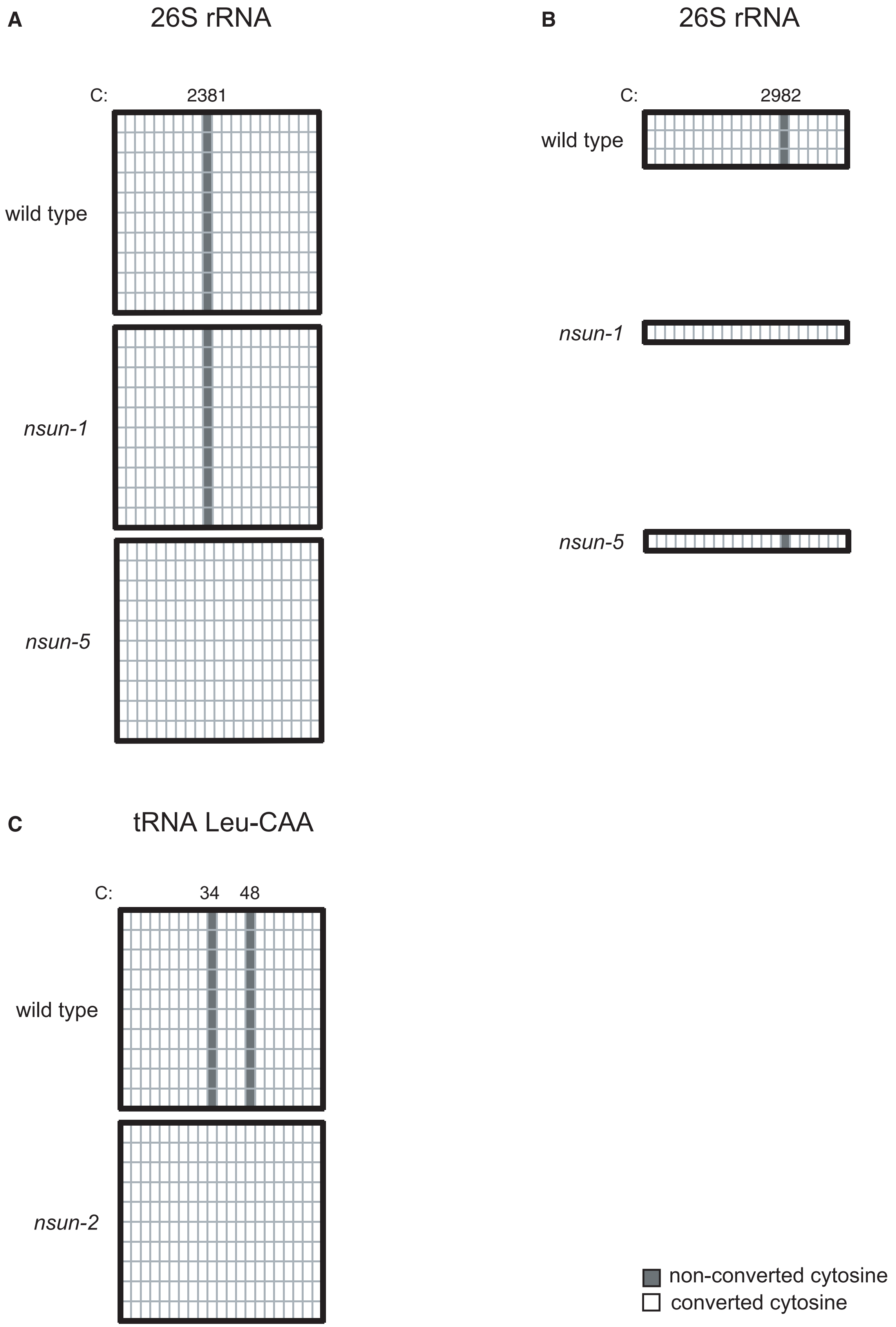

Figure EV1.

 © 2021 The Authors

Figure EV2. Related to Fig 2. Predicted secondary structures of m^5C-methylated ncRNAs.

Red dot indicates the methylated position. Structures predicted by the Predict a Secondary Structure Web Server (David Mathews Lab, University of Rochester) as the lowest free energy structures generated using default data.

© 2021 The Authors

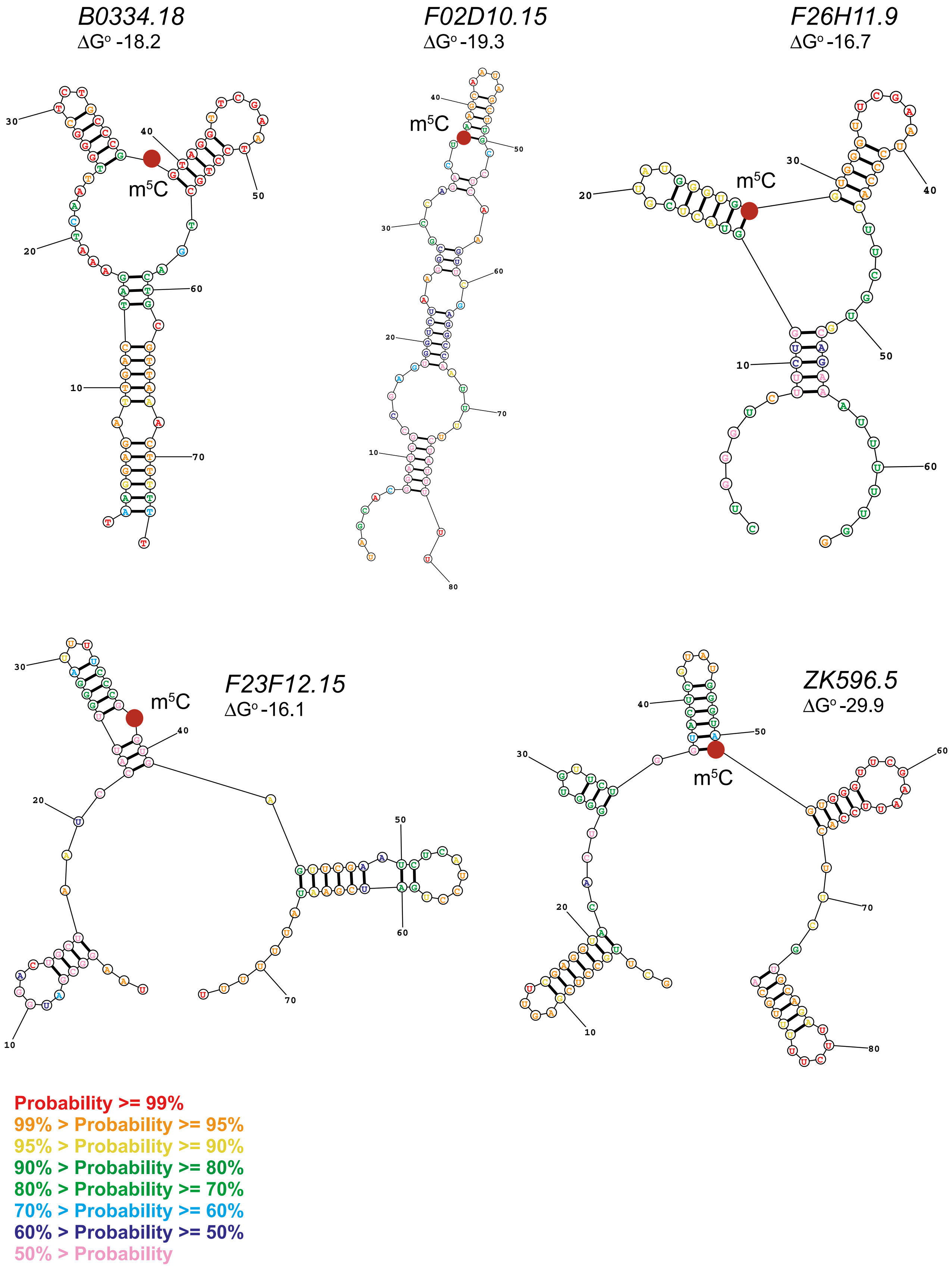

Figure EV2.

© 2021 The Authors

Figure EV3. Related to Fig 5. Differentially transcribed and translated genes upon loss of m^{5}C at different temperatures.

A, B Heat maps and gene ontology enrichment (biological process) analysis for the comparison between wild-type and noNSUN samples. Panel (A) shows RNA-seq (scaled normalised expression) and panel (B) shows Ribo-seq (scaled normalised RPFs). Sets of significant non-redundant GO terms are clustered according to semantic similarity; size indicates the frequency of the GO term in the underlying database WT = wild type; n = 3 biological replicates.

© 2021 The Authors

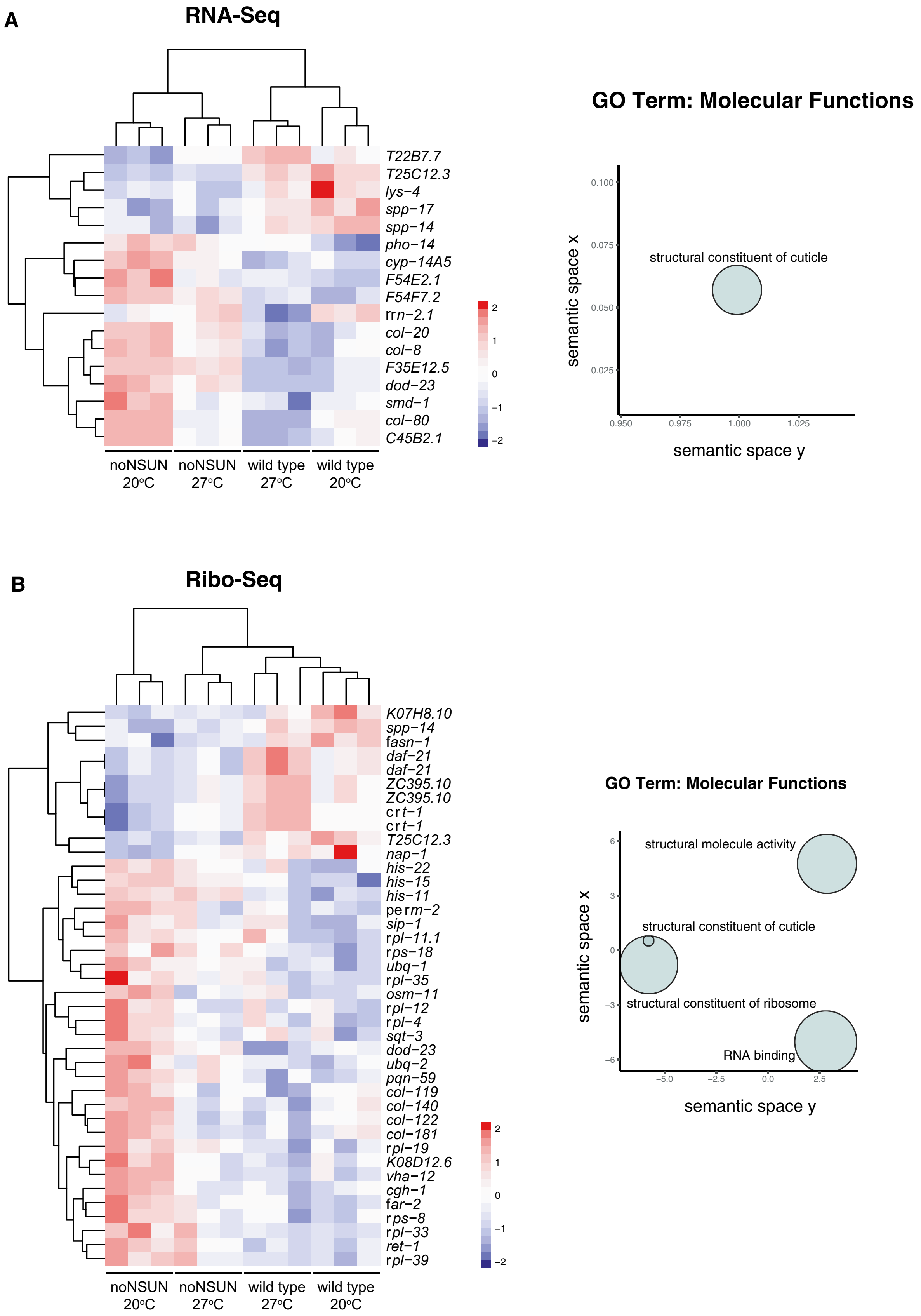

Figure EV3.

 © 2021 The Authors

Figure EV4. Related to Fig 5. Codon occupancy analyses.

A Fold change of P-site codon occupancy in noNSUN over wild-type samples at 20 and 27°C. *P*-values for the fold change occupancy of each codon are indicated in a heat map below the graph, where asterisks indicate statistical significance. $n = 3$ biological replicates.

B Representative examples of UUG codon occupancy in different affected genes. Ribosome-protected fragment counts (RPF) plotted along the gene's CDS. Vertical grey lines indicate UUG codons. $n = 3$ biological replicates.

Data information: In (A), $P < 0.05$, t-test.

© 2021 The Authors

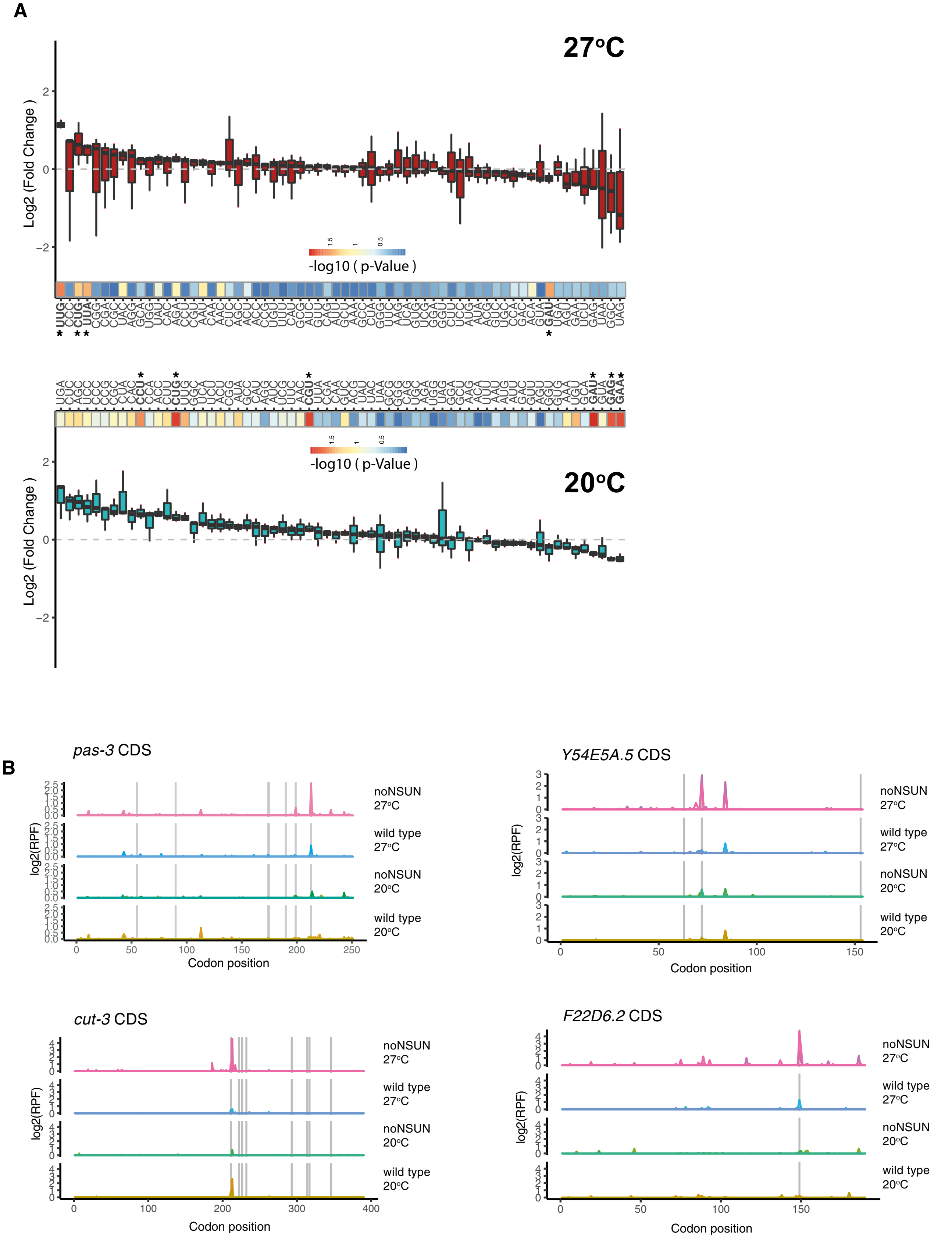

Figure EV4.

 © 2021 The Authors

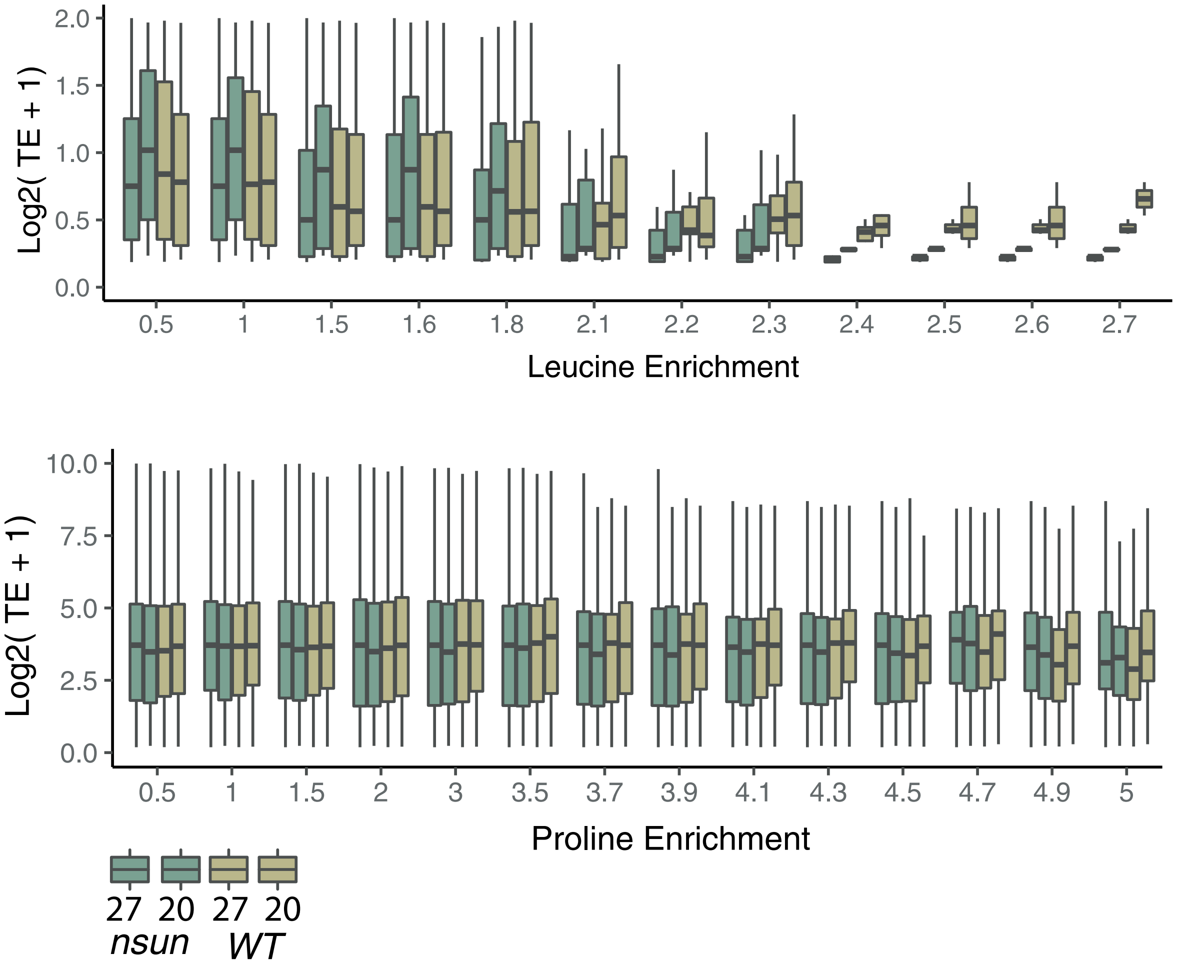

Figure EV5. Related to Fig 5. Translation efficiency of leucine and proline-enriched transcripts.

Translation efficiency of genes with increasing enrichment for leucine (top) or proline codons (bottom). Box plots show the median (central band) and IQR (boxes) ± 1.5 × IQR (whiskers). n = 3 biological replicates.

© 2021 The Authors
